# Controlled Monofunctionalization of Molecular Spherical Nucleic Acids on a Buckminster Fullerene Core

**DOI:** 10.1021/acs.bioconjchem.1c00187

**Published:** 2021-05-16

**Authors:** Vijay Gulumkar, Antti Äärelä, Olli Moisio, Jani Rahkila, Ville Tähtinen, Laura Leimu, Niko Korsoff, Heidi Korhonen, Päivi Poijärvi-Virta, Satu Mikkola, Victor Nesati, Elina Vuorimaa-Laukkanen, Tapani Viitala, Marjo Yliperttula, Anne Roivainen, Pasi Virta

**Affiliations:** † Department of Chemistry, University of Turku, FI-20014 Turku, Finland; § Turku PET Centre, University of Turku, FI-20520 Turku, Finland; ∥ Instrument Centre, Faculty of Science and Engineering, Åbo Akademi University, FI-20500 Åbo, Finland; ⊥ Department of Biologics, Orion Pharma, 20101 Turku, Finland; # Faculty of Engineering and Natural Sciences, Tampere University, FI-33014 Tampere, Finland; ∇ Division of Pharmaceutical Biosciences, Faculty of Pharmacy, University of Helsinki, FI-00014, Helsinki, Finland

## Abstract

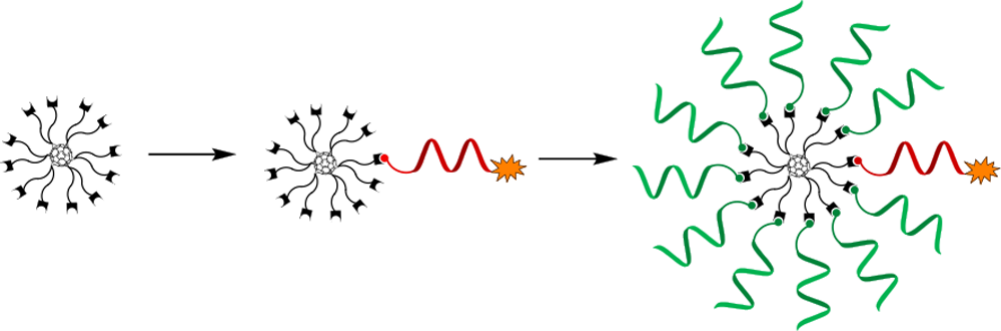

An azide-functionalized 12-armed Buckminster fullerene has been monosubstituted in organic media with a substoichiometric amount of cyclooctyne-modified oligonucleotides. Exposing the intermediate products then to the same reaction (i.e., strain-promoted alkyne–azide cycloaddition, SPAAC) with an excess of slightly different oligonucleotide constituents in an aqueous medium yields molecularly defined monofunctionalized spherical nucleic acids (SNAs). This procedure offers a controlled synthesis scheme in which one oligonucleotide arm can be functionalized with labels or other conjugate groups (1,4,7,10-tetraazacyclododecane-1,4,7,10-tetraacetic acid, DOTA, and Alexa-488 demonstrated), whereas the rest of the 11 arms can be left unmodified or modified by other conjugate groups in order to decorate the SNAs’ outer sphere. Extra attention has been paid to the homogeneity and authenticity of the C_60_-azide scaffold used for the assembly of full-armed SNAs.

## Introduction

Spherical nucleic acids (SNAs, introduced originally by the Chad Mirkin laboratory) consist of an appropriate core (gold, silica, liposomes, proteins) and densely packed oligonucleotide (ON) chains.^1−7^ They share many beneficial properties that overcome some of the major shortcomings perceived for therapeutic ONs: they have efficient free cellular uptake via class A scavenger receptor-mediated endocytosis (which correlates with the density and chemistry of the component ONs),^8,9^ they have muted innate immune responses and resistance to nuclease degradation (due to steric reasons), and they are large enough to avoid renal clearance. The cellular uptake occurs via an endosomal pathway,^8−10^ and SNAs are able to silence target RNAs via steric blocking, once they enter into the cytoplasm.^11^ Also, small interfering RNAs (siRNAs) can work in spherical formulation. Dicer is able to cleave siRNAs from SNAs and release them for the canonical RNA interference pathway.^12^ Most of the reported SNAs are polydisperse structures. The polydispersity may be a shortcoming as the data shows a similar behavior, but still a population of particles lacking thorough characterization of the molecules exists, something that is readily accessible for covalent ON conjugates. Recently, a molecular SNA, based on a Buckminster C_60_-fullerene core, has been described.^13^ This structure, consisting of antisense ON sequences on the C_60_ core, was dense enough to induce scavenger receptor-mediated endocytosis. The internalization of these particles to breast cancer (MCF7) cells was determined to be ca. 500-fold compared to the free component ONs. Regulation of protein expression by an antisense ON that targeted human epidermal growth factor receptor 2 (HER2) mRNA transcripts was also demonstrated.

Our interest in molecularly defined SNAs is to apply them as delivery vehicles together with the covalent conjugation strategy. The rationale of this idea is that radial formulation could be a simple option to emphasize the ligand-specific effect on the outer sphere of the SNAs and at the same time hide the unfavorable distribution properties of negatively charged ONs.^14−17^ For the monitoring of cellular uptake and biodistribution of these decorated SNAs, appropriate labeling is needed, which may cause a misleading distribution and cellular delivery of the actual structure (if all arms are labeled). Therefore, to keep the label effect minimal, an established method that allows controlled monofunctionalization of the SNAs is valuable. Furthermore, the controlled monofunctionalization can be utilized to integrate SNAs specifically with other delivery vehicles. In the present study, an azide-functionalized 12-armed Buckminster fullerene (**1**) is exposed to a substoichiometric amount of cyclooctyne-modified and -labeled (DOTA and Alexa 488) ONs, which gave the monofunctionalized fullerene in relatively high yields. The isolated intermediate products were then exposed to an excess of slightly different ON constituents in an aqueous medium, which gave the monolabeled full-armed SNAs. This two-step process was noticed to be crucial, not only for the controlled assembly but also for the preparation of the C_60_-based SNAs in more general, as the solubility properties of the lipophilic C_60_ core and hydrophilic ONs severely retard the full decoration in one reaction medium only. Reverse-phase high-performance liquid chromatography (RP-HPLC), native polyacrylamide gel electrophoresis (PAGE), capillary electrophoresis (CE), MS spectroscopy, dynamic light scattering (DLS), and size exclusion chromatography equipped with a multiple-angle light-scattering detector (SEC-MALS) were used to analyze the end products. We also paid extra attention to the homogeneity and authenticity of the initial C_60_-azide scaffold (**1**) as it is readily contaminated by a hardly distinguishable azide-C_60_ [3 + 2] cycloaddition side product that would hamper the assembly, purification, and identification of the target SNAs. Overall, this procedure allows a controlled synthesis scheme in which one of the ON arms of SNAs can be selectively functionalized with labels or other conjugate groups. In one case, d-galactose-conjugated ONs were used to decorate the outer sphere. In addition, radiolabeling of a DOTA-labeled SNA has been demonstrated.

## Results and Discussion

### Synthesis and Purification of the C_60_-Azide Core (**1**)

**Scheme 1:**
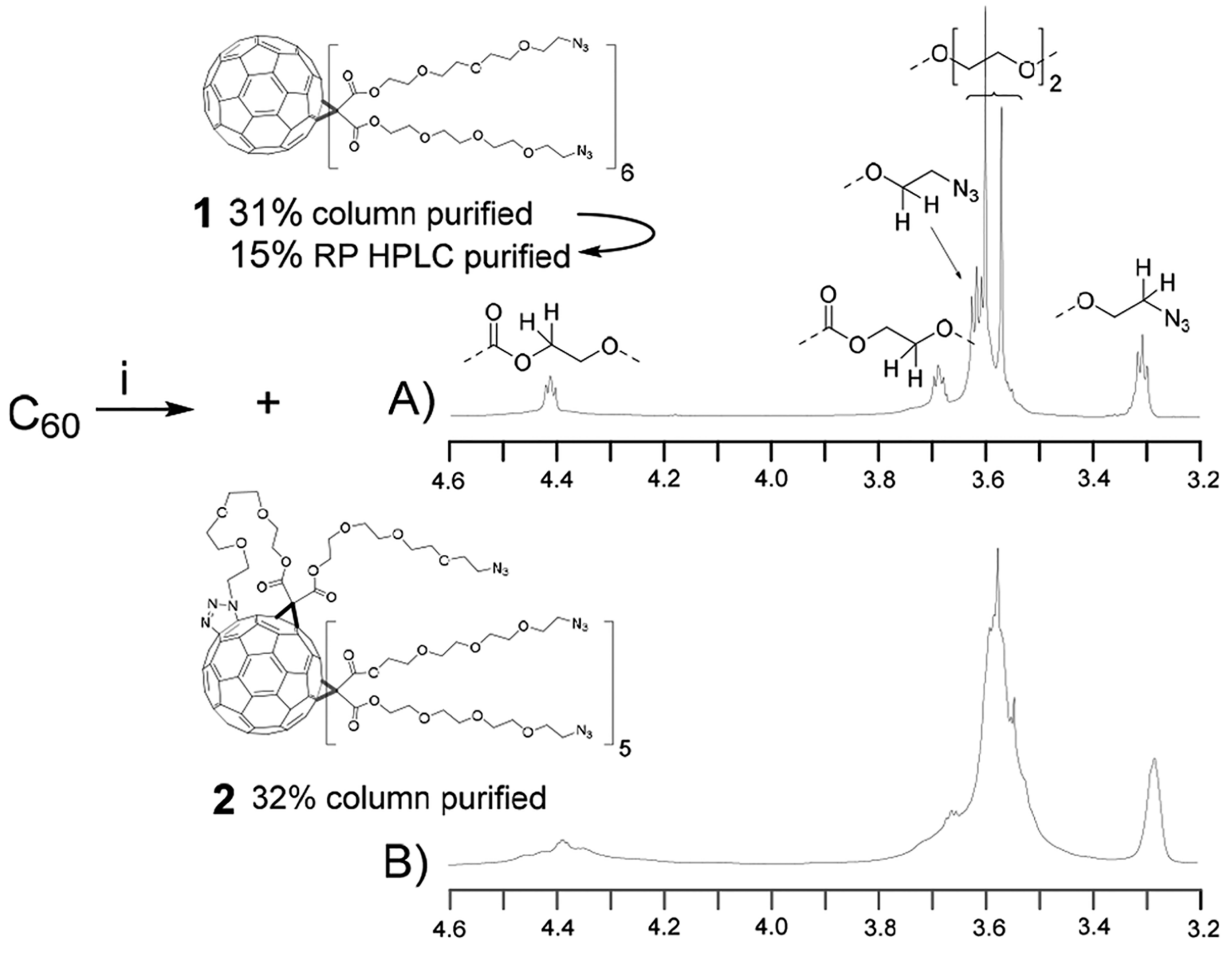
^1^H NMR (500 MHz, CDCl_3_) Spectrum of **1** (A) and **2** (B) Conditions: (i) bis(2-(2-(2-(2-azidoethoxy)ethoxy)ethoxy)ethyl)malonate, CBr_4_, 1,8-diazabicyclo[5,4,0]undec-7-ene (DBU), *o*-dichlorobenzene under argon, 3 days at room temperature.

**Scheme 2:**
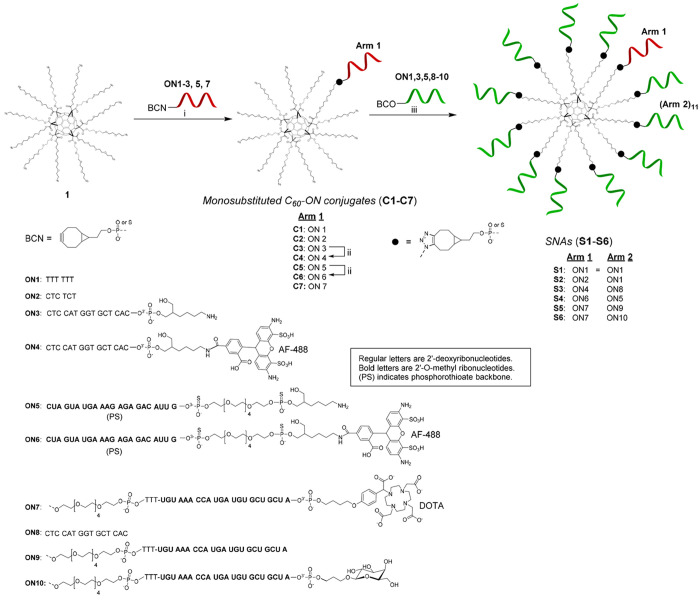
Conditions: (i) BCN-modified oligonucleotide **1** (5 equiv) in DMSO, overnight at 25 °C, (ii) AF-488 NHS ester, 0.1 M sodium borate (pH 8.5), overnight at 25 °C, (iii) **C1–C7**, BCN-modified oligonucleotide (1.2 equiv/arm) in aqueous 1.5 M NaCl, 3 days at 25 °C.

The C_60_-azide scaffold (**1**) was synthesized following a previously published procedure (Scheme 1).^13^ However, in our hands, Bingel’s cyclopropanation^18,19^ between Buckminster fullerene (C_60_) and bis(2-(2-(2-(2-azidoethoxy)ethoxy)ethoxy)ethyl)malonate gave a mixture of compounds (**1** and **2**, ca. 1:1, n/n) with equal molecular masses and similar NMR data (**2** with markedly broader resonances, A vs B in Scheme 1). Repeated column chromatography and RP-HPLC purification were needed to obtain the homogenized **1** in 15% overall isolated yield, which was used for the preparation of SNAs. In order to provide further understanding of the products’ identity and applicability for the SNAs’ assembly, preliminary SPAAC trials were carried out: Both **1** and **2** were exposed to an excess of bicyclo[6.1.0]non-4-yn-9-ylmethanol- and 5′-2-(bicyclo[6.1.0]non-4-yn-9-yl)ethylphosphate (BCN)-modified T_6_ sequence (**ON1**, following the two-step process in Scheme 2). MS (ESI-TOF) analysis verified that all 12 arms of **1** could be readily functionalized, but reactions with **2** stacked to undecafunctionalized products (Figures S6 and S7). ^1^H–^15^N heteronuclear multiple bond correlation (HMBC) analysis was used to further verify the authenticity of **2**, which revealed that part of the nitrogen signals was characteristic to triazol and not entirely to alkylazide (Figures S8 and S9). The fact that the [3 + 2] cycloaddition occurred upon Bingel’s cyclopropanation is understandable, as this reaction has been used to functionalize the C_60_ core in very similar conditions.^20−24^ For further evidence, ad hoc-synthesized triazolino fullerenes were synthesized by treating C_60_ with 2-(2-(2-(2-azidoethoxy)ethoxy)ethoxy)ethanol. The NMR signals of the triazolino fullerenes (di-, tri-, and tetrafunctionalized products obtained, Scheme S1) were comparable to trace signals of **2**.

### Synthesis of Oligonucleotides

For the assembly of SNAs (**S1–S6**), BCN-modified ONs (**ON1**–**ON3**, **ON5**, and **ON7**–**ON10**, Scheme 2) were synthesized using an automated DNA/RNA synthesizer. A standard phosphoramidite coupling cycle and commercially available 2′-*O*-methylribonucleotide and 2′-deoxyribonucleotide building blocks were used for the assembly. 3-Phenyl 1,2,4-dithiazoline-5-one (POS) was used as a sulfurization reagent for the synthesis of **ON5**. Our previously reported customized solid supports^25,26^ were utilized for the synthesis of appropriately 3′-modified ONs: **ON7** and **ON10** with a 1,4,7,10-tetraazacyclododecane-1,4,7,10-tetraacetic acid (DOTA) and a d-galactose moiety, respectively. While **ON1** and **ON2** are short sequences, preliminarily used to demonstrate the SNA’s assembly (**S1** and **S2**), **ON3**–**ON10** are biologically active sequences. **ON3** (and **ON4**) is an antisense sequence that targets HER2 mRNA transcripts. Its biological activity in SNA formulation has previously been demonstrated.^13^ The phosphorothioate (PS) sequence of **ON5** (and **ON6**) is a splice switching ON that prevents expression of an androgenic receptor variant (AR-V7) in prostate cancer cells.^27^ The 2′-*O*-methylated sequence found in **ON7**, **ON9**, and **ON10** is complementary to micro-RNA 15b that is involved in hepatocyte apoptosis.^28,29^ We have previously ^68^Ga labeled this same ON and its glycoconjugates and studied their biodistribution by in vivo positron emission tomography/computed tomography (PET/CT) imaging.^30−32^

### Controlled Assembly of Monofunctionalized SNAs on the C_60_-Azide Core

**Figure 1:**
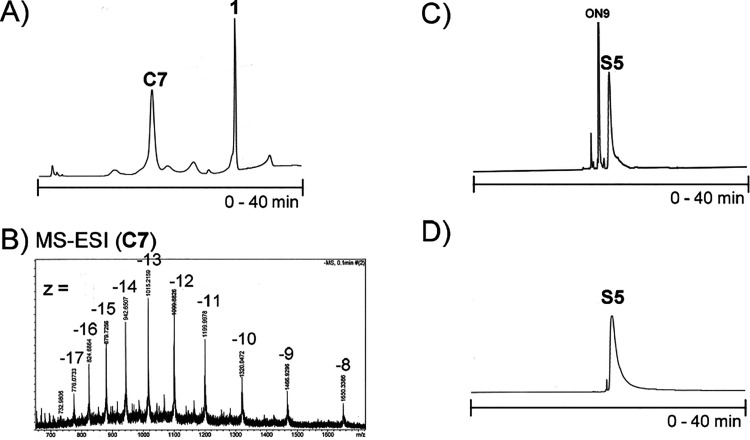
(A and C) Examples of RP-HPLC profiles of crude product (**C7** and **S5**) mixtures. (B) Example of the MS-ESI spectrum of purified monosubstituted C_60_-ON-conjugate (**C7**). (D) Example of the RP-HPLC profile of purified SNA (**S5**).

In initial trials, the C_60_-azide core **1** was dissolved in a minimum volume of DMSO and treated with an excess (>12 equiv) of BCN-modified oligonucleotides in an aqueous solution containing 1.5 M NaCl.^13^ However, the drastically different solubility properties between the lipophilic C_60_-azide core (**1**) and the hydrophilic ONs retarded the SPAAC conjugation, and complex mixtures of products were obtained (reactions using different DMSO–H_2_O ratios, different spacers between the ONs and the core, BCN- vs dibenzocyclooctyne-modified ONs, and different temperature were attempted). This guided us to try a two-step process in which **1** was first conjugated with ONs in DMSO, and once the partially functionalized more hydrophilic intermediate products were obtained, the reaction was changed to an aqueous medium to yield full-armed SNAs. Interestingly, monofunctionalization proceeded in DMSO (Figure 1A, Scheme S2) with a reasonable excess of **1**, which could be utilized for the controlled assembly of heteroantennary SNAs (**S1**--**S6**, Scheme 2). In optimized conditions (Scheme 2), BCN-modified ONs (**ON1–3**, **ON5**, and **ON7**) were treated with 5 equiv of **1** in DMSO to yield monofunctionalized C_60_–ON conjugates (**C1**–**C3**, **C5**, and **C7**) in relatively high RP-HPLC-isolated yields (45–50%). The remaining excess of **1** could be reisolated (cf. Figure 1A) and reused. The amino-modified conjugates (**C3** and **C5**) were labeled with Alexa-488-*N*-hydroxysuccinimide (NHS) ester, and the conjugates (**C1**, **C2**, **C4**, **C6**, and **C7**) were then dissolved in aqueous 1.5 M NaCl solution and mixed with a slight excess (12 equiv) of BCN–ONs (**ON1**, **ON5**, and **ON8**–**ON10**).^33−35^ After incubation for 2 days at room temperature, one additional equivalent of BCN–ONs was added to confirm the completion of the full decoration (in overall 1.2 equiv/arm). The reaction mixtures were then incubated one more day (3 days total) and subjected as such to RP-HPLC (Figure 1C, Scheme S2). The obtained SNAs (**S1**–**S6**) were isolated in 40–57% yield according to UV absorbance at 260 nm (**S1**, **S2**, **S5**, and **S6**) and 488 nm (**S3** and **S4**).

### Characterization of the SNAs

**Figure 2:**
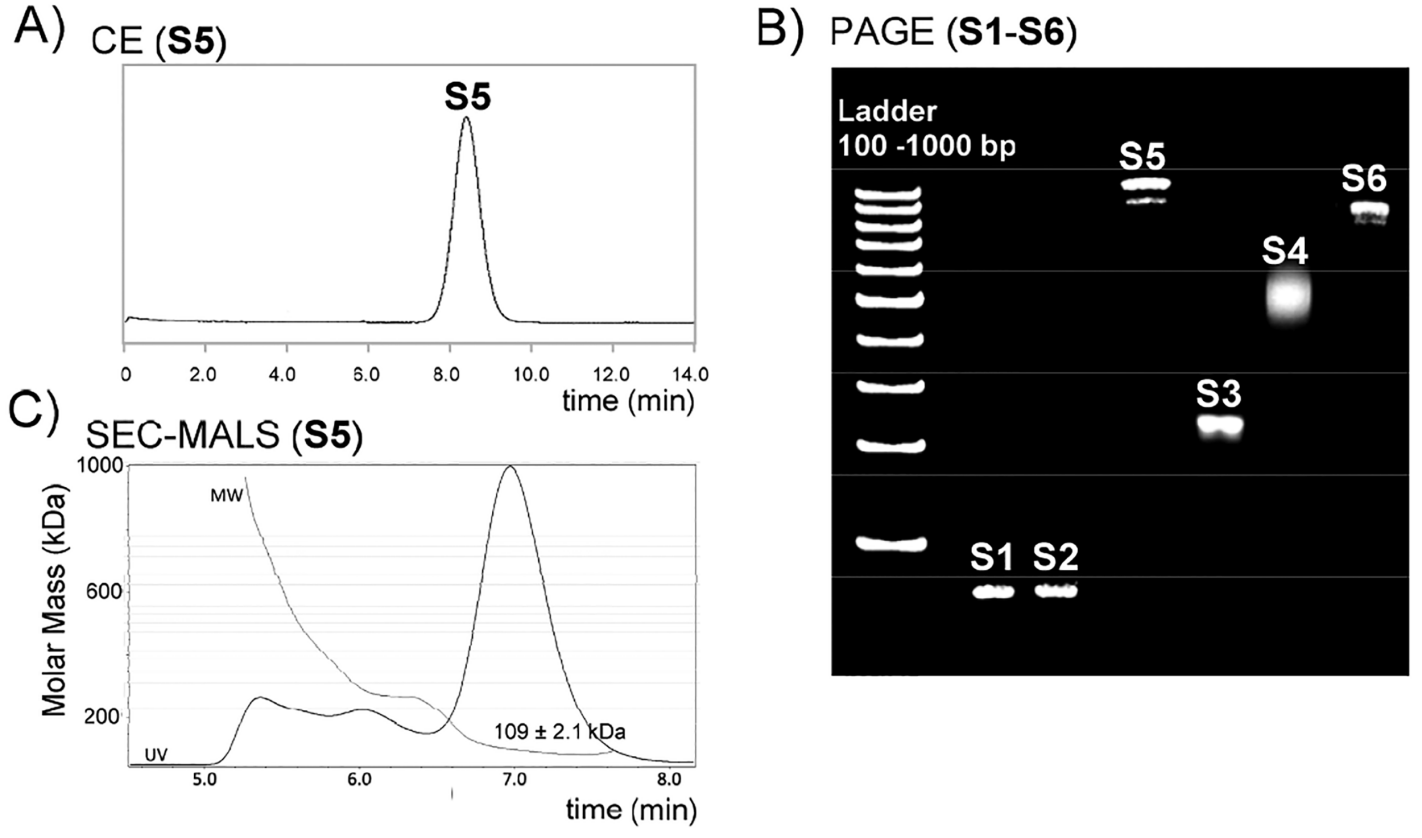
(A) Example of capillary electrophoregrams (CE) of purified SNA (**S5**). (B) Polyacrylamide gel electrophoregrams (PAGE) of purified SNAs (**S1**–**S6**). (C) Example of the SEC-MALS profile of purified SNA (**S5**) used to evaluate the molecular mass. For the conditions, see Experimental Section.

The homogeneity and identity of the RP-HPLC-isolated SNAs were evaluated by PAGE, CE, SEC-MALS, and MS-ESI spectroscopy. As seen on PAGE (Figure 2B), each SNA resulted in a distinct and relatively sharp band. The stereoisomeric phosphorothioate backbone of **S4** caused a blurred band compared to other SNAs. On the electrophoregrams of larger SNAs (**S5** and **S6**), faster eluting trace products (<5% of the total intensity) could be additionally observed that indicated incomplete decoration. Fractionation by RP-HPLC (Figure 1C) did not affect the results on PAGE, neither did the prolonged reaction time nor a higher excess of BCN-ONs (**ON9** and **ON10**) used for the SPAAC conjugation. Despite these traces of side products (most likely 11-armed SNAs), the overall purity of the assembled SNAs after single RP-HPLC purification proved relatively high on PAGE. Next, the applicability of CE to evaluate the homogeneity of the SNAs was demonstrated (Figure 2A and Scheme S2). CE could not discriminate traces of incomplete (11-armed) products from the full-armed SNAs, but it proved to be a valuable tool to confirm the absence of smaller component ONs. SEC-MALS is a common technique employed to estimate the homogeneity, aggregation tendency, and molecular weight of biomolecules.^36^ This is particularly useful for large molecular weight compounds (>100 kDa), characterization of which is often hardly accessible by MS spectroscopy. This was the case also with SNAs. Acceptable *m*/*z* data (a spectrometer equipped with a hybrid quadrupole orbitrap and nano ESI ionization was used) could be obtained for small model SNAs **S1** and **S2** (Figure S12), whereas humps of overlapping multiply charged ion patterns, unsuitable for reliable MS characterization, were obtained for **S3**–**S6**. In fact, even **S1** and **S2** were prone to form stable multiple sodium adducts (Figure S12), and the observed molecular masses were 0.1 and 0.2 kDa higher compared to the calculated values (Table 1, entries 1 and 2). Therefore, we applied SEC-MALS to estimate the molecular weights of **S3**–**S5**. The samples of **S3**–**S5** were eluted with 150 mM phosphate buffer (pH 7) through a 300 Å, 2.7 μm, 4.6 × 300 mm SEC column. As seen in Figure 2C and Figure S12, each SNA resulted in a major peak (retention time ca. 7 min) that represented the 70–80% mass fraction of the sample. The MALS-based estimation of the molecular weights extracted from the major peaks matched relatively well with the expected calculated values (Table 1, entries 3–6). It may be worth mentioning that the errors of the observed molecular masses were less than the molecular mass of the component ONs. In each case, faster eluting fractions (retention time ca. 5.0–6.5 min) were observed also, the molecular weight of which (>200 kDa) may be attributed to aggregation of the SNAs (Figure 2C and Figure S12). Together with the molecular masses obtained for small model SNAs **S1** and **S2** and the SEC-MALS-based characterization of SNAs **S3**–**S6**, the authenticity of the products could be verified. Finally, the hydrodynamic diameter of the SNAs was determined by DLS. The diameters (ranging from 9.2 to 21.4 nm, Table 1) of **S1**–**S6** correlated with the lengths of the component ONs.

**Table 1:** Molecular Masses of SNAs **S1–S6**

SNA	calculated molecular mass (kDa)	observed molecular mass (kDa)	hydrodynamic size (nm)
**S1**	27.6	27.8^a^	9.2 ± 1.2
**S2**	27.6	27.7^a^	9.6 ± 2.5
**S3**	61.2	62.6 ± 0.4^b^	11.8 ± 0.6
**S4**	107.3	106.8 ± 3.6^b^	16.2 ± 0.1
**S5**	109.9	109.3 ± 2.1^b^	19.0 ± 2.0
**S6**	113.2	107.4 ± 2.3^b^	21.4 ± 0.8

a A hybrid quadrupole-orbitrap spectrometer with nano ESI-ionization was used for mass analysis (cf. Figure S12).

b The values were obtained by SEC-MALS-based estimation of the molecular mass (cf. Figure 2C).

### Melting Analysis (*T*_m_) and Titration of SNA with a Complementary RNA Strand

**Figure 3:**
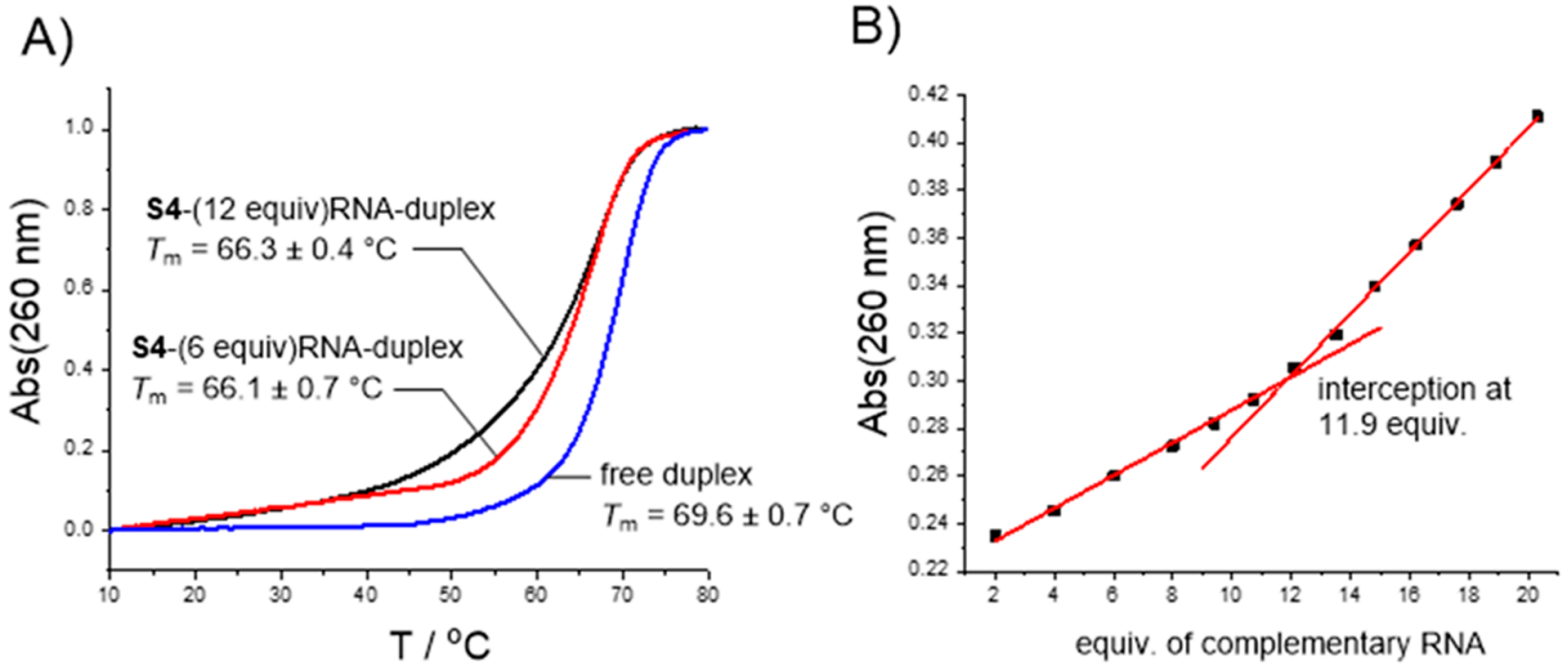
Melting profile analysis and titration of **S4** with complementary RNA: C AAU GUC UCU CUU UCA UAC UAG. For the formation of free duplex CUA GUA UGA AAG AGA GAC AUU G (2′-*O*-methyl RNA phosphorothioate) was used.

In order to evaluate the hybridization properties of the C_60_-based SNAs, UV-melting profile experiments (*T*_m_) and titration of **S4** with a short model sequence of AR-V7 pre-mRNA were carried out. The **S4**–RNA duplex resulted in a −3 °C decrease in the *T*_m_ value when compared to the corresponding free duplex (Figure 3A). Furthermore, gentler melting profiles were observed, which was more obvious, when fully hybridized **S4** was compared to a partially hybridized one (12 vs 6 equiv of complementary strands). This indicates electrostatic repulsion/steric crowding between the duplexes on fully loaded SNA (**S4** + 12 equiv of RNA). This observation is consistent with the previous findings in which a retarded loading of siRNAs onto SNAs has been observed.^12,37−39^ However, it is notable that the fully loaded SNA (**S4** + 12 equiv of RNA) was virtually stable below the physiological temperature and the observed “’premature’” partial denaturation occurred at a higher temperature. Titration of **S4** with the same complementary RNA verified the correct stoichiometry of the melting profiles (Figure 3B; note, the concentration of **S4** was determined according to the Alexa content). Gradual addition of the complementary RNA strand increased the overall absorbance with a constant slope (hypochromic effect due to the hybridization compensates for the increased absorbance), and a turning point of the slope was observed once the amount of the complementary RNA exceeded the fully occupied SNA. The observed turning point at 11.9 equivalents matched well with the correct 12-armed SNA structure.

### Radiolabeling

**Figure 4:**
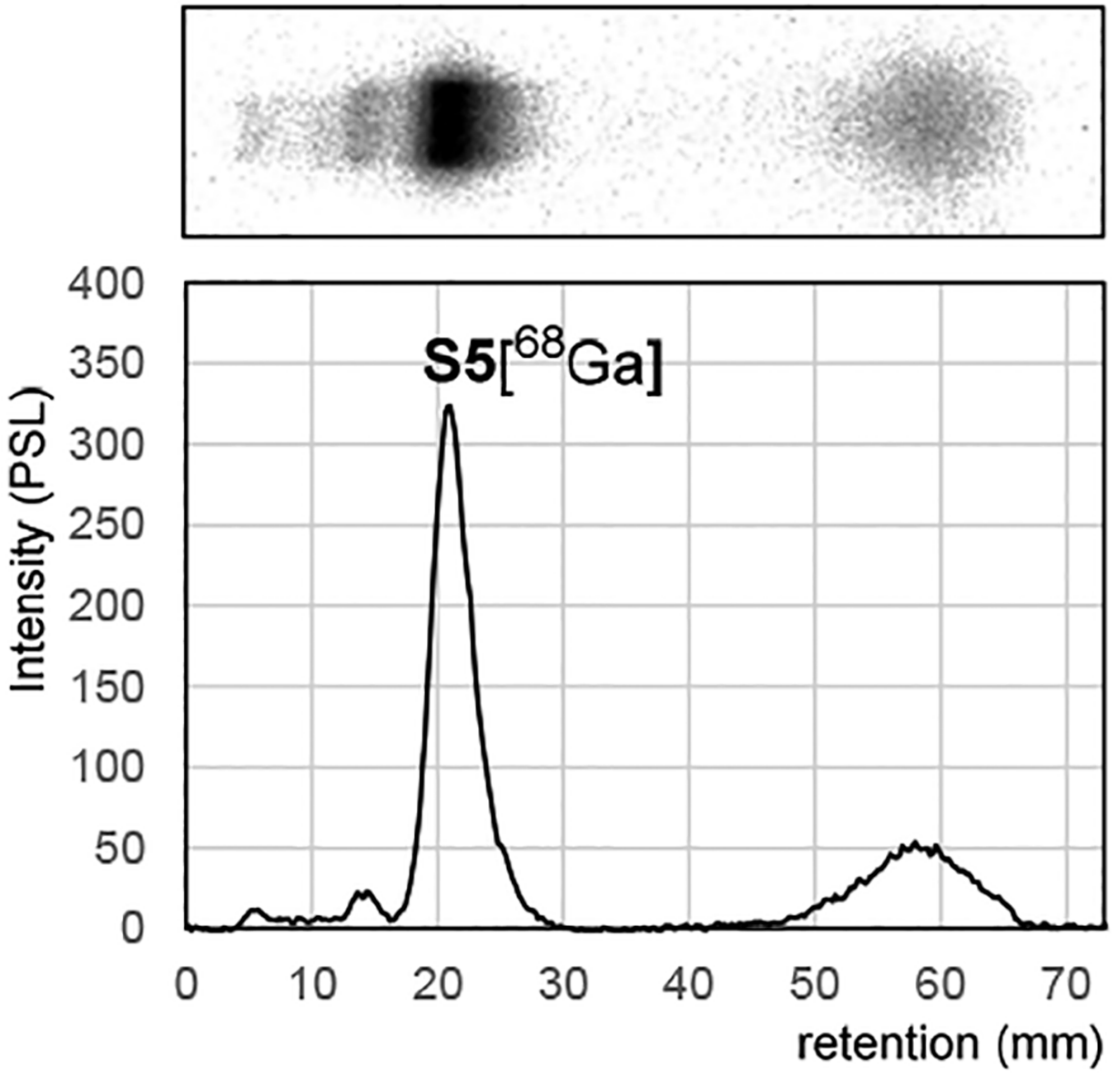
Representative polyacrylamide gel electrophoresis (PAGE) autoradiography image and quantification of radiolabeled SNA **S5[**^**68**^**Ga]**.

To evaluate the applicability of DOTA as a ^68^Ga-chelating agent on SNAs, **S5** was used as a model in preliminary radiolabeling experiments. With these small-scale trials (1–2 nmol of **S5**) we also tried to find conditions that minimize precursor loading at the expense of yield. Radiochemical yields of up to 68 MBq were achieved (24% decay corrected yield). Radiochemical purity was measured at up to 69% as measured by PAGE and 73% by ultrafiltration (Figure 4). We observed that size exclusion purification of the reaction mixture could not sufficiently remove all unbound ^68^Ga, even when two successive column purifications were used. Also, commonly used solid-phase extraction columns with C8, C18, and hydrophobic lipophilic balance (HLB) solid phases were attempted. Due to the difficulty of separating unbound ^68^Ga and the low performance of the size exclusion purification, we suspected unspecific binding of ^68^Ga to the SNA structure. This was confirmed by incubating the end product (**S5[**^**68**^**Ga]**) in 50 mM ethylenediaminetetraacetic acid/phosphate-buffered saline (EDTA/PBS) at pH 7.4 in 37 °C. As expected, we observed a further 10% increase in the unbound activity fraction after 1 h EDTA challenge, as measured by ultrafiltration. This relatively stable unspecific ^68^Ga binding to the densely packed ON construct is in agreement with the behavior of the SNAs in MS in which relatively stable multiple sodium adducts were observed (Table 1, entries 1 and 2). Although the small-scale (1–2 nmol of precursor **S5** loading) experiments did not produce acceptable purities, valuable information was obtained, which will aid future in vivo PET/CT imaging studies of SNAs. Due to the unspecific ^68^Ga binding observed, an indirect labeling method, such as a click reaction with a reactive agent prelabeled with either ^68^Ga or ^18^F, may prove a more suitable choice for SNAs.^40,41^

## Conclusion

A two-step procedure that allows controlled assembly of monofunctionalized C_60_-based SNAs has been described. This preliminary data opens the door for controlled decoration of SNAs with labels or tissue/organ-specific ligands and possibility integrating them specifically to other delivery vehicles. Despite the multicomponent SPAAC-based assembly and two RP-HPLC purifications, the overall yield of these SNAs proved to be relatively high, ca. 20–30%. We also paid extra attention to the homogeneity of the C_60_-azide scaffold (**1**). **1** was noticed to be readily contaminated by a hardly distinguishable azide-C_60_ [3 + 2] cycloaddition side product (**2**) that would hamper the assembly, purification, and identification of the SNAs. The homogeneity and authenticity of the SNAs were evaluated by various methods, including RP-HPLC, native PAGE, CE, SEC-MALS, and MS-ESI spectroscopy. While PAGE was a superior technique to evaluate the homogeneity of these C_60_-based SNAs, SEC-MALS could be used for rough evaluation of the molecular weights. Preliminary radiolabeling experiments suggested that the chelation-based techniques should be replaced by a covalent radiolabeling strategy to prevent unspecific metal ion (^68^Ga) binding to the densely packed ON shell of these nanoparticles. The described procedure with detailed analytical control of each single step will expectedly promote design of new molecularly defined monofunctionalized SNAs, which may find interesting therapeutic and diagnostic applications.

## Experimental Section

### Synthesis and Purification of the C_60_-Azide Core (**1**)

**1** was synthesized by Bingel cyclopropanation following the previously published procedure:^13^ Buckminster fullerene C_60_ (0.40 g, 0.56 mmol) was dissolved in dry and degassed (oxygen removed by bubbling with argon) *o*-dichlorobenzene (140 mL). Bis(2-(2-(2-(2-azidoethoxy)ethoxy)ethoxy)ethyl)malonate (2.8 g, 5.6 mmol, 10 equiv), CBr_4_ (19 g, 56 mmol, 100 equiv), and 1,8-diazabicyclo[5,4,0]undec-7-ene (DBU, 1.7 mL, 11 mmol, 20 equiv) were added, and the mixture was stirred 3 days at room temperature under argon. The mixture was purified by silica gel column chromatography (pore size 60 Å, 230–400 mesh particle size, 40–63 μm particle size, isocratic elution with 2% MeOH in dichloromethane, twice) to yield the desired product **1** (0.66 g, 32%) and a near identically behaving side product **2** (0.65 g, 31%, for further characterization, see Figures S2–S9). A sample (30 mg) of column-purified **1** was further purified by RP-HPLC to yield homogenized **1** (7 mg, 23%, overall yield 15%), which was used for the preparation of SNAs. **1**: ^1^H NMR (500 MHz, CDCl_3_) δ 4.43 (t, 24H, *J* = 4.1 Hz), 3.75 (t, 24H, *J* = 4.1 Hz), 3.69 (t, 24H, *J* = 4.3 Hz), 3.67 (s, 48H), 3.64 (s, 48H), 3.40 (t, 24H, *J* = 4.2 Hz); ^13^C NMR (125 MHz, CDCl_3_) δ 163.5, 145.8, 141.0, 70.7, 70.0, 69.0, 68.6, 65.8, 50.7, 45.2; MS (ESI-TOF) molecular mass for C_174_H_192_N_36_O_60_Na_3_ 3816.6, found 3816.4 (calculated from [(M + 3Na)/3]^3+^.

### Synthesis of C_60_-ON Conjugates **C1–C3**, **C5**, and **C7**

General procedure: BCN-modified oligonucleotide (**ON1–3**, **ON5**, or **ON7**, 0.1 μmol in 60 μL of H_2_O) was slowly added to a mixture of C_60_ core **1** (0.5 μmol in 540 μL of DMSO) in a microcentrifuge tube. The reaction mixture was gently shaken overnight at room temperature and subjected to RP-HPLC (Figure 1A and Scheme S2). An analytical RP-HPLC column (250 × 4.6 mm, 5 μm), a gradient elution from 40% to 100% MeCN in 50 mmol L^–1^ triethylammonium acetate over 30 min, and detection at 260 nm were applied. The C_60_–ON conjugate (**C1**–**C7**) and unreacted fullerene core **1** were collected individually and lyophilized to dryness. The authenticity of the products was verified by MS (ESI-TOF) (Figure 1B and Figure S11). Isolated yields (45–50%, only slight differences in yields between the different conjugates observed) of **C1**–**C7** were determined by UV absorbance at 260 nm.

### Synthesis of Alexa-488-Labeled Conjugates **C4** and **C6**

AF488 NHS ester (0.4 μmol in 6 μL of DMSO) was added to a buffered mixture of C_60_–ON conjugate (50 nmol of **C3** or **C5** in 60 μL of 0.1 M sodium borate, pH 8.5). The reaction mixture was gently shaken overnight at room temperature and subjected to RP-HPLC. An analytical RP-HPLC column (250 × 4.6 mm, 5 μm), a gradient elution from 40% to 100% MeCN in 50 mmol L^–1^ triethylammonium acetate over 30 min, and detection at 260 nm were used. The product fractions were collected and lyophilized to dryness. The authenticity of the products (**C4** and **C6**) was verified by MS (ESI-TOF) (Figure S11). Isolated yields (**C4**, 32%; **C6**, 62%) of the products were determined by UV absorbance at 488 nm.

### Assembly of SNAs (**S1–S6**)

General procedure: C_60_–ON conjugate (**C1**–**C7**, 20 nmol in 55 μL of H_2_O) was mixed with BCN–ON (**ON1**, **ON3**, **ON5**, and **ON8**–**ON**10****, 240 nmol in 145 μL of H_2_O), and 100 μL of 4.65 M NaCl was added. The reaction mixture was gently shaken for 2 days at room temperature, and then one additional equivalent of BCN–ON was added. The mixture was then incubated one more day (72 h total using 1.2 equiv of BCN–ON/azide arm) and subjected to RP-HPLC. An analytical RP-HPLC column Phenomenex, Aeris 3.6 μm WIDEPORE XB-C18 200 Å, 150 × 4.6 mm, a linear gradient from 5% to 60% MeCN in 50 mmol L ^–1^ triethylammonium acetate over 40 min, a flow rate of 1.0 mL min ^–1^, and detection at 260 nm were used for purification (Figure 1C and Scheme S2). The product (**S1**–**S6**) fractions were collected and lyophilized to dryness. Isolated yields (40–57%) of the products were determined by UV absorbance at 260 nm (**S1**, **S2**, **S5**, and **S6**) and 488 nm (**S3** and **S4**). The obtained SNAs were characterized by MS-ESI (equipped with a hybrid quadrupole orbitrap and nano-ESI ionization) (**S1** and **S2**) and SEC-MALS (**S3**–**S6**) (Table 1, Figure 2C, and Figure S12). For the homogeneity and particle size evaluation of **S1**–**S6**, see the PAGE (Figure 2B), CE (Figure 2A and Scheme S2), SEC-MALS (Figure 2C, Scheme S2), and DLS experiments described below.

### PAGE Analysis of SNAs

Native 6% Tris base, boric acid, EDTA, and acrylamide (TBE) gel were used to check SNAs’ purity. A precast gel cover (10 cm × 10 cm in size, Thermo Fisher Scientific) was fixed into a vertical electrophoresis chamber, and the running buffer (90 mM Tris, 90 mM borate, and 2 mM EDTA, 8.3 pH) was filled into the chamber. SNA samples (5 μL of 0.1 μM SNAs mixed with 5 μL of TBE sample buffer) and a DNA ladder (100, 200···1000 bp; note, the ladder is just used to confirm the quality and comparability of the runs and cannot be used for size evaluation of the SNAs) were loaded and electrophoresed at constant 200 V (45 mA) for approximately 30 min. After completion of electrophoresis, gel was removed from the chamber and the SNA bands were monitored either directly by UV or after staining by SYBRTM Gold Nucleic Acid Stain (Thermo Fisher Scientific).

### Capillary Electrophoresis Experiments

Samples were analyzed by capillary zone electrophoresis in a fused silica capillary of 75 μm i.d. and 57 cm effective length. The background electrolyte was 0.3 M citrate buffer, pH 3.1. A voltage of 15 kV and pressure of 0.3 PSI were applied. UV detection at λ = 260 nm was used.

### SEC-MALS Experiments

SEC-MALS was performed using an Agilent Technologies 1260 Infinity II HPLC system (sampler, pump, and UV–vis detector) equipped with a Wyatt Technologies miniDAWN light scattering detector and Wyatt Technologies Optilab refractive index detector. An Agilent AdvanceBio SEC 300 Å 2.7 μm 4.6 × 300 mm column and 150 mM sodium phosphate, pH 7.0, as mobile phase eluting at a rate of 0.2 mL min^–1^ and run time of 20 min were used for each experiment. For each run, 4 μL of sample with a SNA concentration of 1 mg mL^–1^ in Milli-Q water was loaded onto the pre-equilibrated column. Detector signals were aligned with a bovine serum albumin (BSA) standard, which was analyzed prior to SNA samples. The RI and MALS signals were used for the MW calculations using an average refractive index increment (d*n*/d*c*) of 0.1703 mL/g.

### Melting Experiments (*T*_m_) and Titration of **S4** with a Complementary RNA

The melting curves (absorbance vs temperature) were measured at 260 nm on a UV–vis spectrometer equipped with a multiple cell holder and a Peltier temperature controller. The temperature was changed at a rate of 0.5 °C min^–1^ between 10 and 80 °C. The measurements were performed in 10 mmol L^–1^ sodium cacodylate (pH 7.0) with 0.1 mol L^–1^ NaCl and 1.0 μmol L^–1^ ON. *T*_m_ values were determined as the maximum of the first derivative of the melting curve. The UV titration of **S4** (absorbance vs equivalents of complementary RNA oligonucleotide added) was performed in 10 mmol L^–1^ sodium cacodylate (pH 7.0) with 0.1 μmol L^–1^ NaCl and 63 nmol L^–1^**S4**. RNA oligonucleotide with a complementary sequence to **S4** was added gradually in the solution, and the total absorbance at 260 nm was monitored with an UV–vis spectrometer. The sequence of complementary RNA was C AAU GUC UCU CUU UCA UAC UAG. For the formation of free duplex, CUA GUA UGA AAG AGA GAC AUU G (2′-*O*-methyl RNA phosphorothioate) was used.

### DLS Experiments

The size of the SNAs was measured at room temperature using a Zetasizer Nano ZS90 (Malvern Instruments Ltd., UK). The settings and conditions for the measurements were as follows: material Protein (RI, 1.450; absorption, 0.001), dispersant water (viscosity, 0,8872 cP; RI, 1.330) temperature was 20 °C, and equilibration time was 60 s. Each sample (10 μg of SNA in 100 μL of aqueous 10 mmol L^–1^ PBS, 2.7 mmol L^–1^ M KCl, 0.137 mol L^–1^ NaCl, pH 7.4) was measured three times.

### Radiolabeling Experiments

[^68^Ga]GaCl_3_ was eluted from an IGG-100 ^68^Ge/^68^Ga generator with 0.1 M HCl through a Strata SCX cartridge into a waste container. The cartridge was then eluted with 300 μL of 1.0 M sodium chloride/0.1 M HCl solution, and an aliquot (200 μL) was transferred to a reaction vial preloaded with a mixture of HEPES (12 mg) and **S5** (1–2 nmol) in 50 μL of water. Gentisic acid (10 μL, 0.1 M in water) was added as a radical scavenger to counter possible radiolysis. The reaction mixture was then incubated at 70 °C for 15 min. The mixture was cooled on ice after incubation and purified using consecutive Illustra NAP-5 and NAP-10 size-exclusion columns (Cytiva, USA) equilibrated with phosphate-buffered saline (PBS, pH 7.4). This afforded the end product formulation in 1.2 mL of phosphate-buffered saline (PBS). Radiochemical purity was determined by native PAGE and ultrafiltration. PAGE was carried out with TBE-buffered 6% polyacrylamide gels in a Biorad miniprotean II system (Bio-Rad Laboratories, Hercules, CA, USA) ran at 250 V. The gels were developed on BAS-TR2025 phosphor imaging plates and analyzed with a BAS-5000 scanner (Fuji, Tokyo, Japan). Ultrafiltration was done in triplicate by loading 0.5 mL, 30 kDa Microcon filters (Millipore, Bedford, MA, USA), with 100 μL of PBS and 1 μL of reaction mixture. The filters were centrifuged three times for 5 min at 14 000*g*, with addition of 100 μL of PBS between spins. The activities of the filter and the filtrate were measured with a 1480 Wizard gamma counter (PerkinElmer/Wallac, Turku, Finland), and the purity was calculated by dividing the filter activity by the total activity.

## Supporting Information Available

Further characterization data of **1** and **2**, monosubstituted C_60_-conjugates **C1**–**C7**, and SNAs (**S1**–**S6**) (PDF)
