# Supplementary material for: Controlled Monofunctionalization of Molecular Spherical Nucleic Acids on a Buckminster Fullerene Core
Source: Bioconjug Chem. 2021 May 16;32(6):1130–8. doi: 10.1021/acs.bioconjchem.1c00187 (PMC8382215; doi:10.1021/acs.bioconjchem.1c00187)
Supplement: Supplementary file 1 — bc1c00187_si_001.pdf [file bc1c00187_si_001.pdf]

Supporting Information to:

Controlled monofunctionalization of molecular spherical nucleic acids on Buckminster fullerene core

Vijay Gulumkar,[a] Antti Äärelä,[a] Olli Moisio,[b] Jani Rahkila,[c] Ville Tähtinen,[a] Laura Leimu,[d] Niko Korsoff,[a] Heidi Korhonen,[a] Päivi Poijärvi-Virta,[a] Satu Mikkola,[a] Victor Nesati,[d] Elina Vuorimaa-Laukkanen,[e] Tapani Viitala,[f] Marjo Yliperttula,[f] Anne Roivainen,[b] Pasi Virta[a],[d]*

[a] *Department of Chemistry, University of Turku, FI-20014 Turku, Finland, E-mail: pamavi@utu.fi*
[b] *Turku PET Centre, University of Turku, FI-20520 Turku, Finland*
[c] *Instrument centre, Faculty of Science and Engineering, Åbo Akademi University, FI-20500 Åbo, Finland*
[d] *Department of Biologics, Orion Pharma, 20101 Turku, Finland*
[e] *Tampere University, Faculty of Engineering and Natural Sciences, FI-33014 Tampere University, Finland*
[f] *Division of Pharmaceutical Biosciences, Faculty of Pharmacy, University of Helsinki, FI-00014, Helsinki, Finland*

Contents

Synthesis of fullerene (C_{60}) core 1 **S2**
Figure S1. A) An RP HPLC profile of silica gel column-purified **1**.
B) RP HPLC profile of homogenized **1** **S2**
Figure S2. ^{1}H NMR (500MHz, $CDCl_3$) and COSY spectra of **1**. **S2**
Figure S3. ^{13}C NMR (125 MHz, CDCl3) and HSQC spectrum of **1.** **S3**
Figure S4. ^{1}H NMR ($CDCl_3$, 500MHz) and COSY spectrum of **2.** **S4**
Figure S5. ^{13}C NMR (125 MHz, $CDCl_3$) and HSQC spectra of **2.** **S5**
Figure S6. MS (ESI-TOF) spectra of **1** and of cyclo addition products of **1** and **2**. **S6**
Figure S7. MS-ESI spectrum of 11-armed model SNA assembled on **2.** **S6**

2D ^{1}H-^{15}N and ^{1}H-^{13}C correlation experiments to verify authenticity of 2 **S7**
Figure S8. ^{1}H-^{15}N HMBC spectra of **2** and *ad hoc* synthesized triazolinofullerene **S8**
Figure S9. ^{1}H-^{13}C- HSQC spectra of **2** and *ad hoc*-synthesized triazolinofullerene **S8**

***Ad hoc*-synthesized triazolinofullerenes** **S9**
Scheme S1. *Ad hoc*-synthesis of triazolino fullerenes. **S9**
Figure S10. MS-ESI (TOF) analysis of *ad hoc*-synthesized triazolinofullerenes **S9**

Synthesis of C_{60}-SNAs S1-S6 **S10**
Scheme S2. Purification scheme of the SNAs' assembly **S10**
Figure S11. MS(ESI-TOF) data of C_{60}-ON-conjugates (**C1**-**C7**) **S11**
Figure S12. A and B) MS-ESI data of C_{60}-SNAs **S1** and **S2**,
C-F) SEC-MALS data of C_{60}-SNAs **S3**-**S6** **S12**

Synthesis of fullerene (C_{60}) core 1

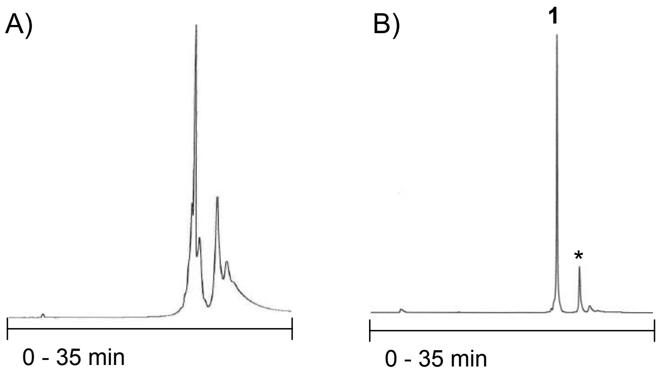

Figure S1. A) An RP HPLC profile of silica gel column-purified **1**. B) RP HPLC profile of homogenized **1**. The minor peak (marked *) is also the product (co-injection of either peak gives exactly the same profile). A gradient elution from 60 to 100% MeCN in H_2O over 25 min., continued then by 100% MeCN, an analytical RP HPLC column (C18, 250 × 5 mm, 4.5 µm).

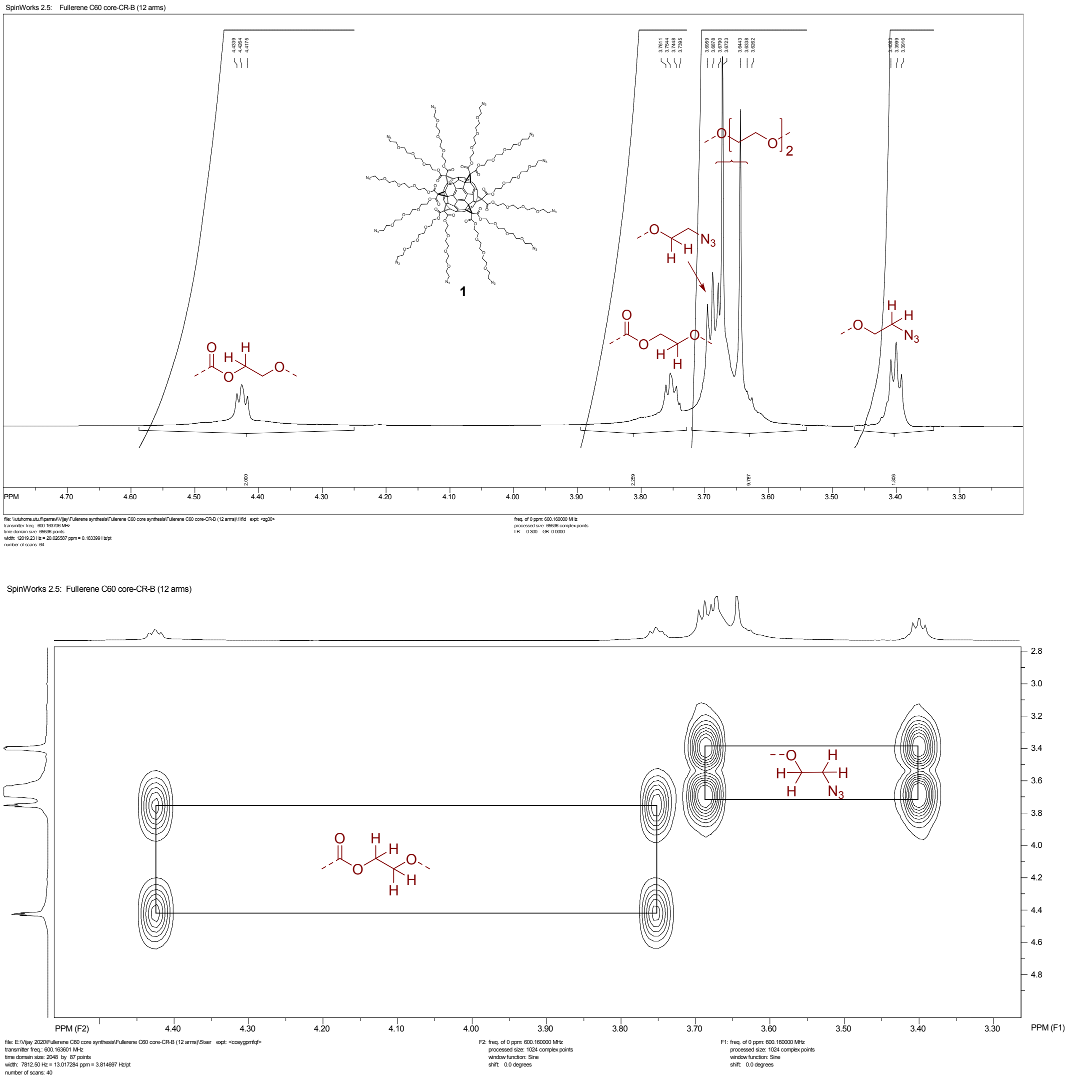

Figure S2. ^{1}H NMR (500MHz, $CDCl_3$) and COSY spectra of **1**.

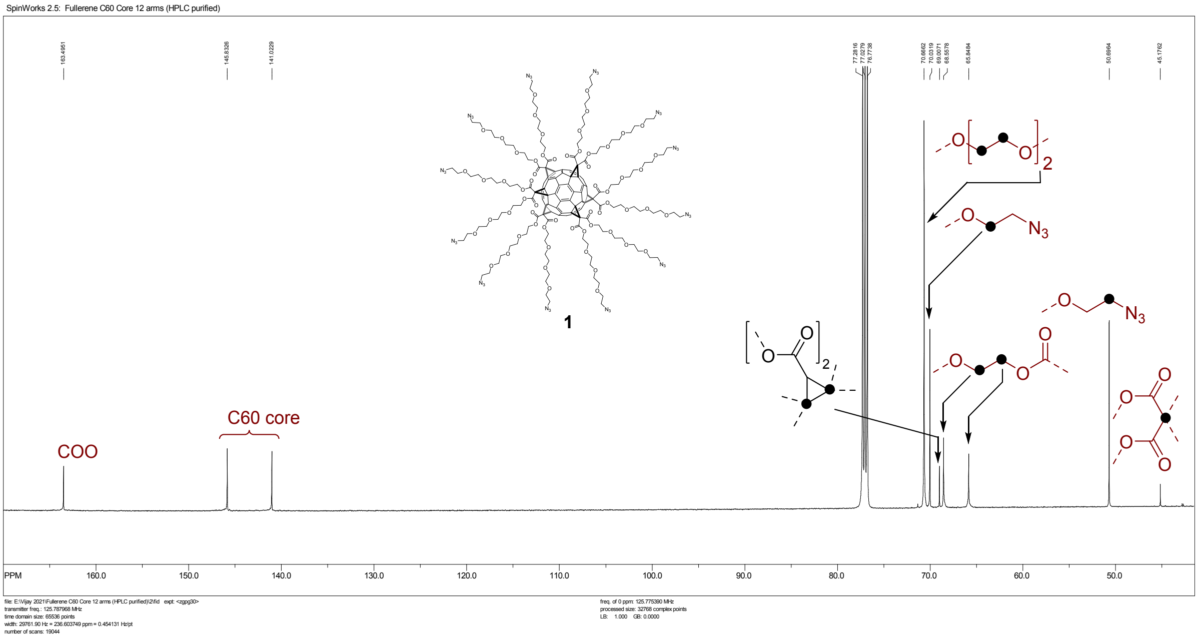

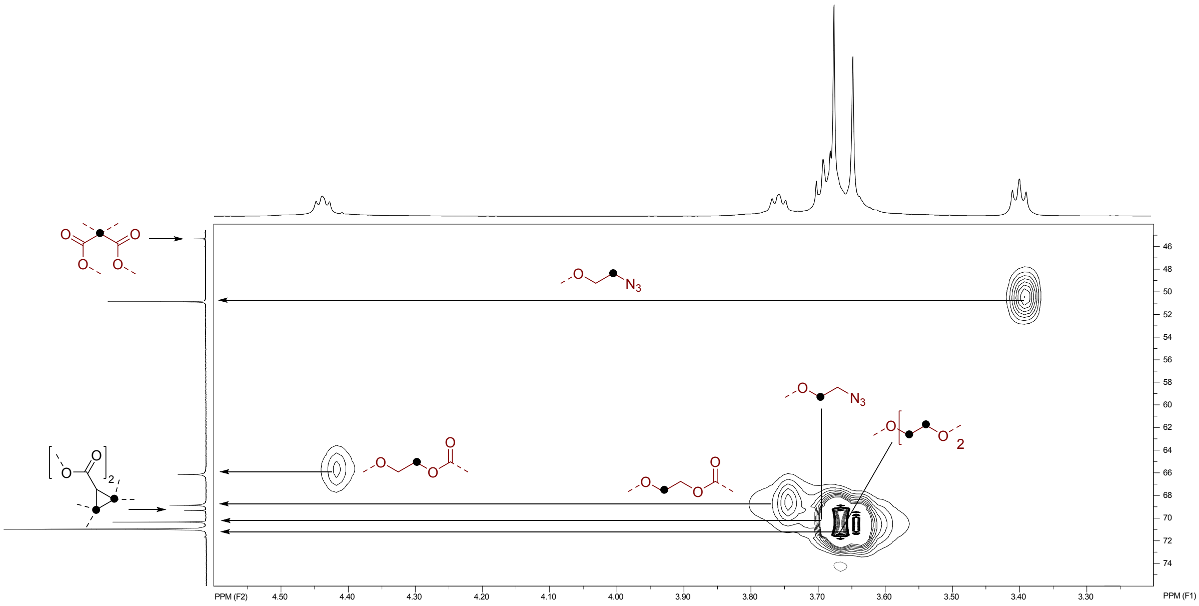

Figure S3. ${}^{13}C$ NMR (125 MHz, CDCl3) and HSQC spectrum of **1.**

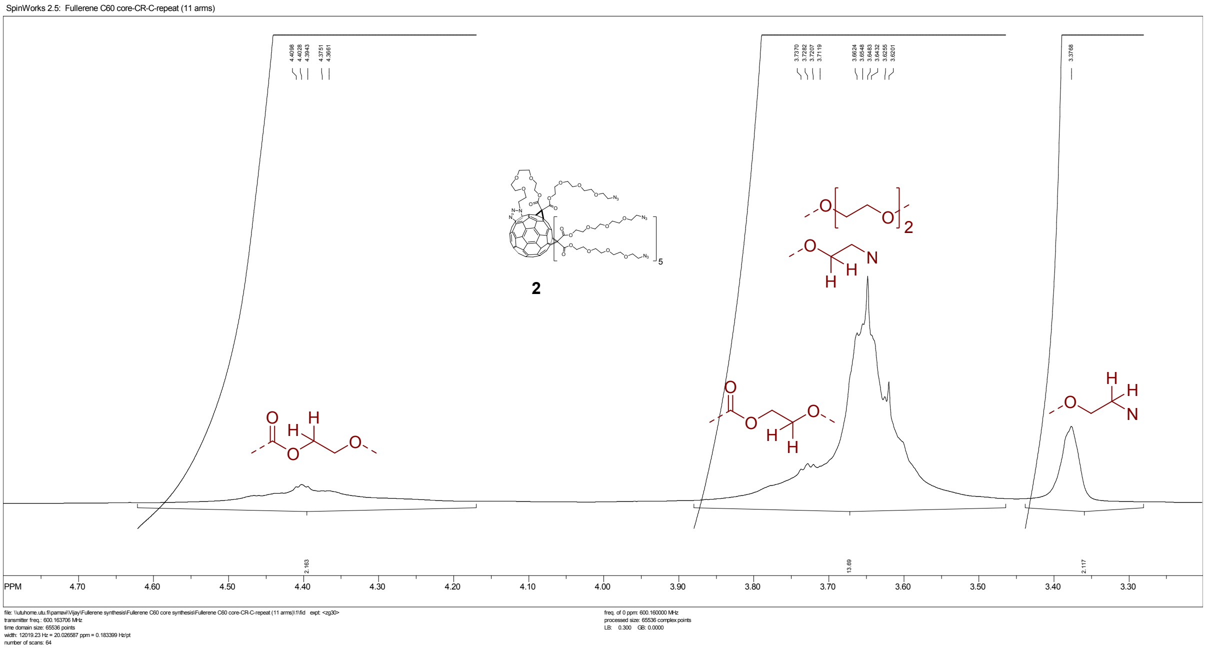

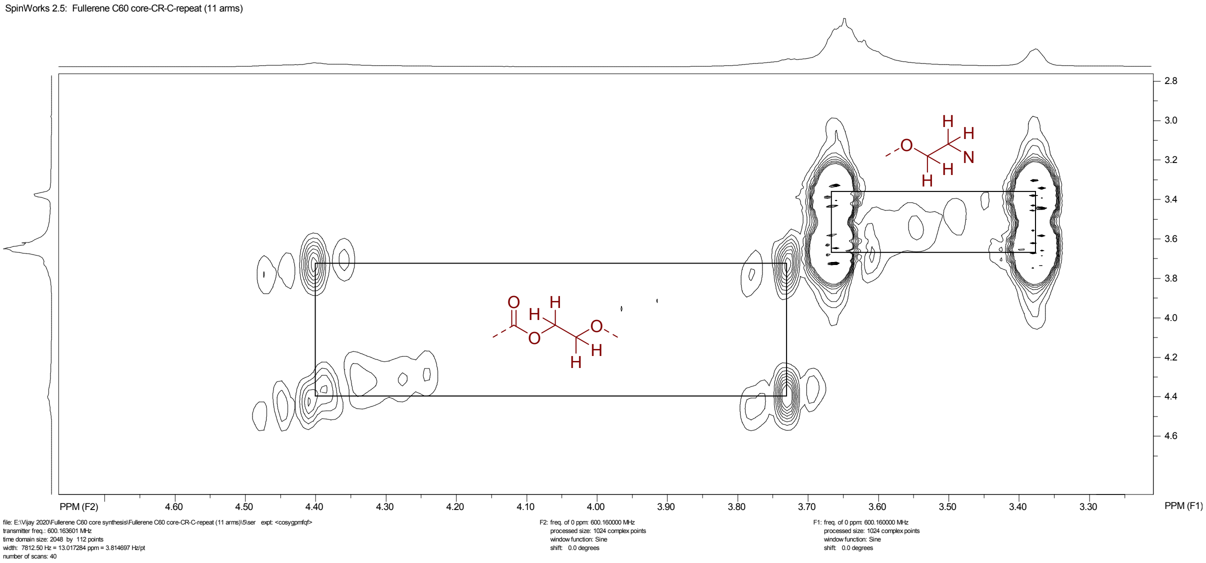

Figure S4. ^{1}H NMR ($CDCl_3$, 500MHz) and COSY spectrum of **2.**

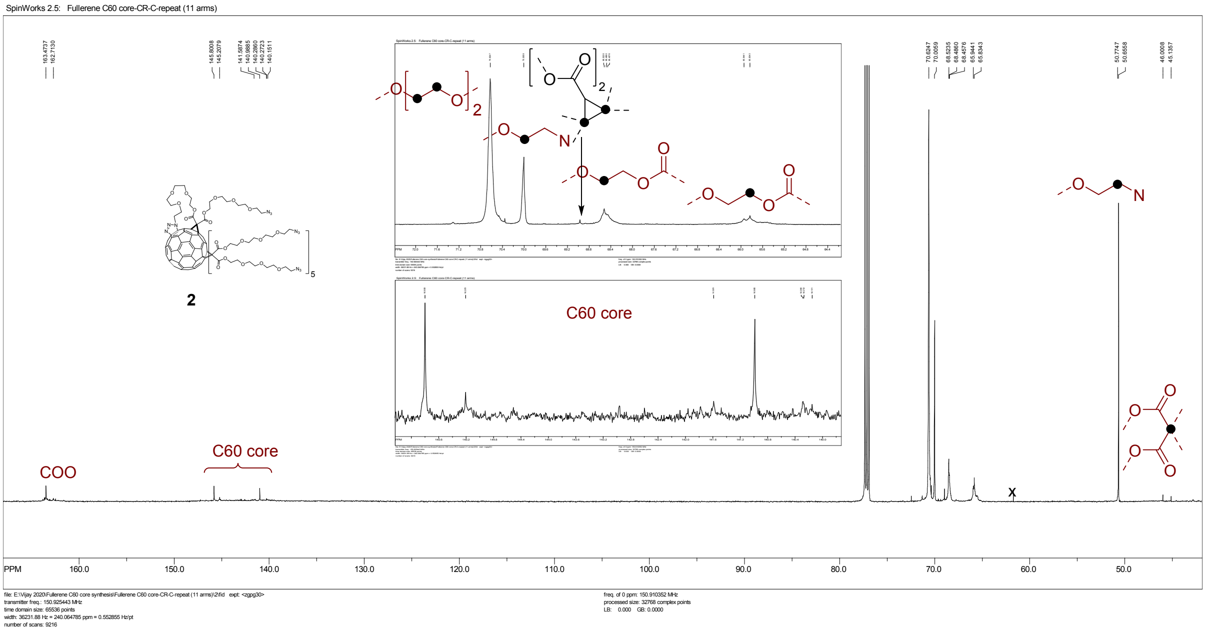

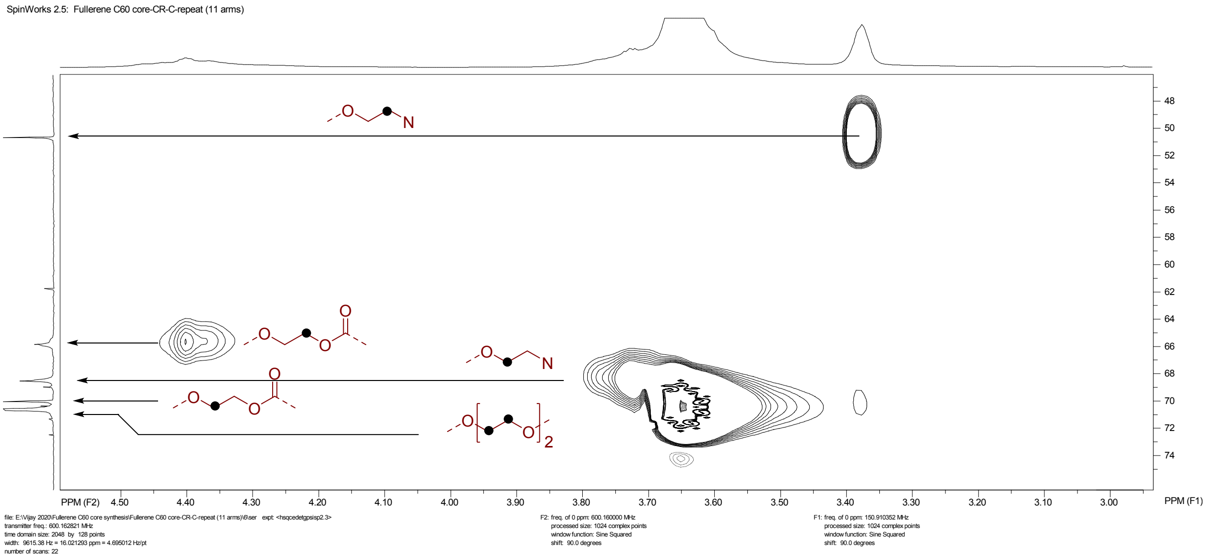

Figure S5. ^{13}C NMR (125 MHz, $CDCl_3$) and HSQC spectra of **2.**

Note: the low intensity of the typical sp^2 carbons and the cyclopropane carbons on the C60 core. Furthermore, some extra signals in the aromatic region can be observed. These indicate distorted symmetricity of the C60 core.

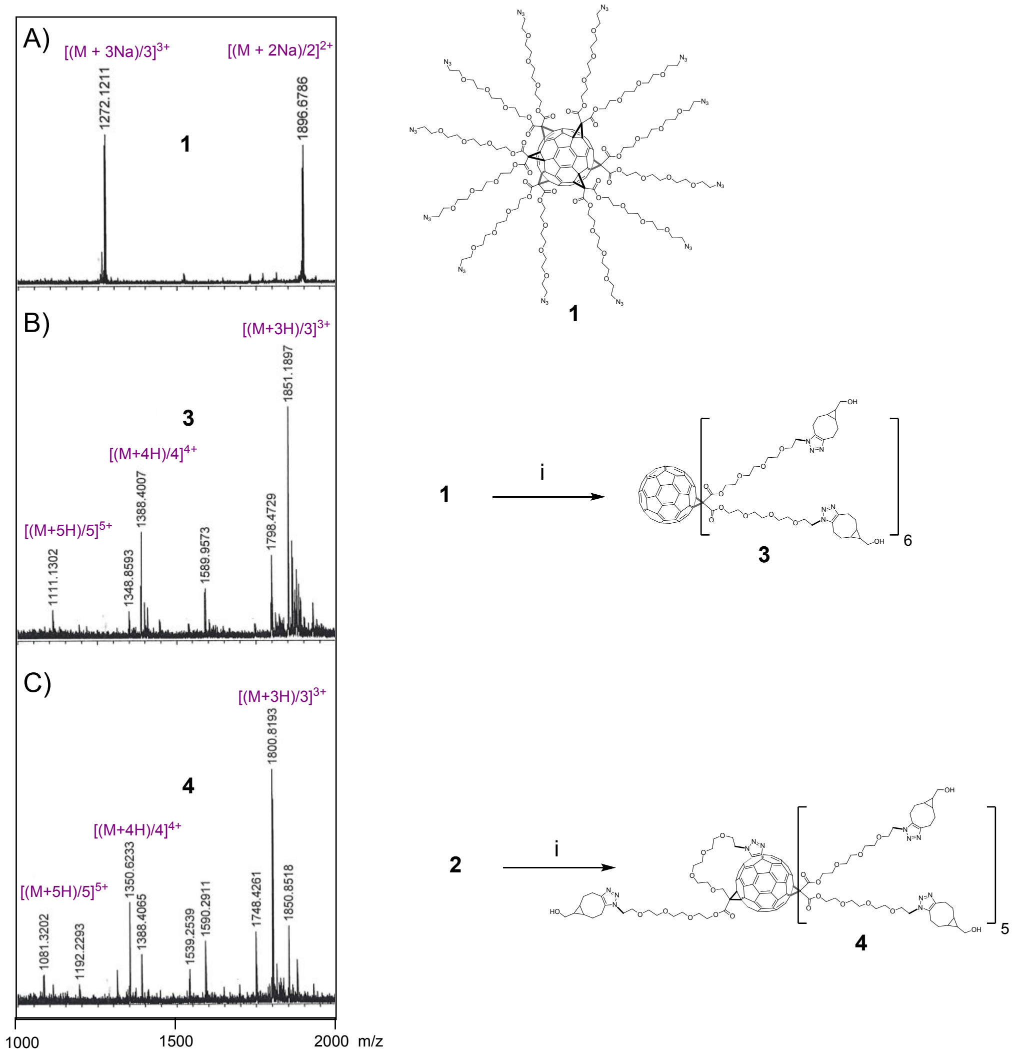

Figure S6. MS (ESI-TOF) spectra of **1** and of cyclo addition products of **1** and **2**. A) homogenized **1** (**2** gave exactly the same mass), B) a product mixture when an analytical sample of **1** was exposed to **i** C) a product mixture when an analytical sample of **2** was exposed to **i**. **i** = an excess of bicyclo[6.1.0]non-4-yn-9-ylmethanol in DMSO, overnight at 55°C.

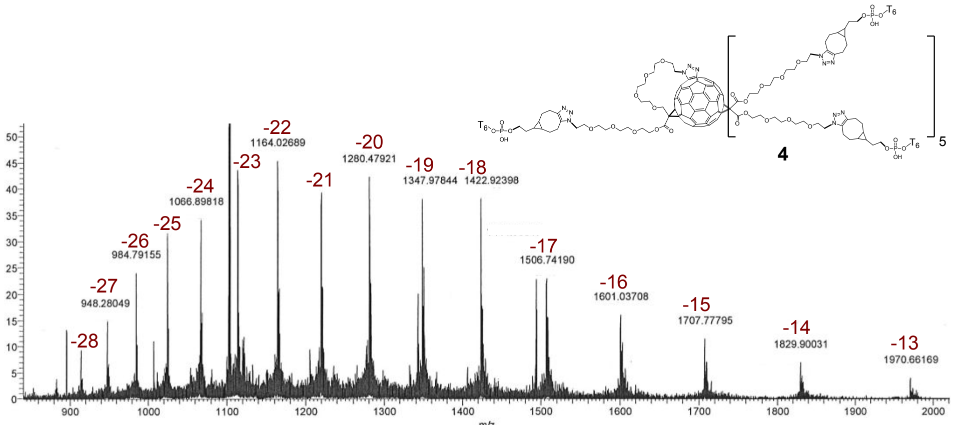

Figure S7. MS-ESI spectrum of 11-armed model SNA assembled on **2**.

2D ^{1}H-^{15}N and ^{1}H-^{13}C correlation experiments to verify authenticity of 2

^{1}H-^{15}N HMBC was used to determine the ^{15}N chemical shifts of **2**. In addition to the expected signals from the azide at 69.0 ppm (N=N=***N***-CH_2-), 210.6 ppm (***N***=N=N-CH_2-) and 248.3 (N=***N***=N-CH_2-),[1] two more ^{15}N signals were found at approximately 195 ppm and 413 ppm (**Figure S8**). These new peaks suggest that one of the azides might have been reacted with the fullerene core to form a triazoline.[2] It may be worth mentioning that the similar conditions, used for the Bingel's cyclopropanaton to obtain **1**, have been reported to yield triazolinofullerenes.[33]

To further confirm the identity of **2**. ^{1}H-, ^{13}C HSQC and HMBC spectra were recorded in order to obtain the ^{13}C chemical shifts of the two methylene groups closest to the triazoline. These were found to be approximately at 46 ppm and 71 ppm for the N-***C***H_2- and N-CH2-***C***H_2- carbons respectively. In particular the chemical shift of the methylene group next to the nitrogen has a significantly different chemical shift compared to the methylene group next to an azide at 50.5 ppm (**Figure S9**). This is in good agreement with similar triazolinofullerenes reported previously.2[,4]

The low concentration of the triazoline compared to the azides complicated complete characterization of **2**. Therefore, triazolino-derivatives of C_{60} were prepared (*ad hoc*) using 2-(2-(2-azidoethoxy)ethoxy)ethoxy)ethanol (Figure S9). The ^{13}C and ^{15}N chemical shifts of the triazolinofullerene were found to be very similar to the ones seen in **2**.

The ^{1}H chemical shift of the methylene group next to the triazoline is somewhat lower field than those observed in the *ad hoc* synthesized triazolinofullerenes (a product mixture). However, the chemical shifts of ^{1}H are generally more sensitive towards the exact environment than ^{13}C and ^{15}N chemical shifts. The general trend is the same though; the ^{1}H chemical shift of the methylene next to the nitrogen is slightly higher field than that of the one next to the oxygen.

From these characterization data, supported by the experimental observations of the reactivity (**1** *vs.* **2**, Figure S6), we can confirm that **2** contains an inactive cyclo addition adduct (i.e. a triazolino group on the fullerene core).

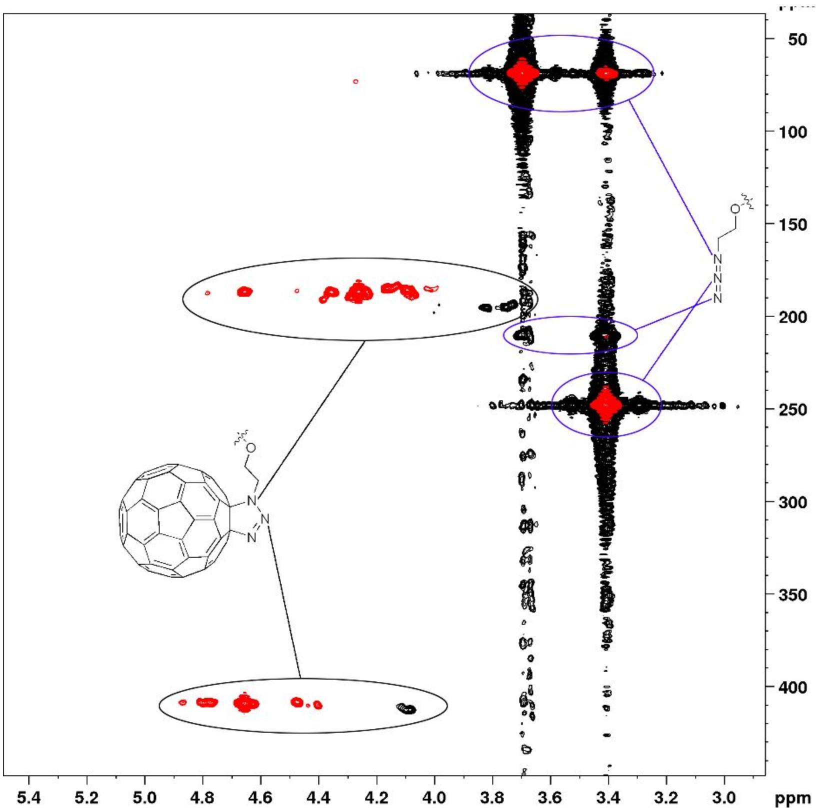

Figure S8. ^{1}H-^{15}N HMBC spectra of **2** (black) and *ad hoc* synthesized triazolinofullerene (red).

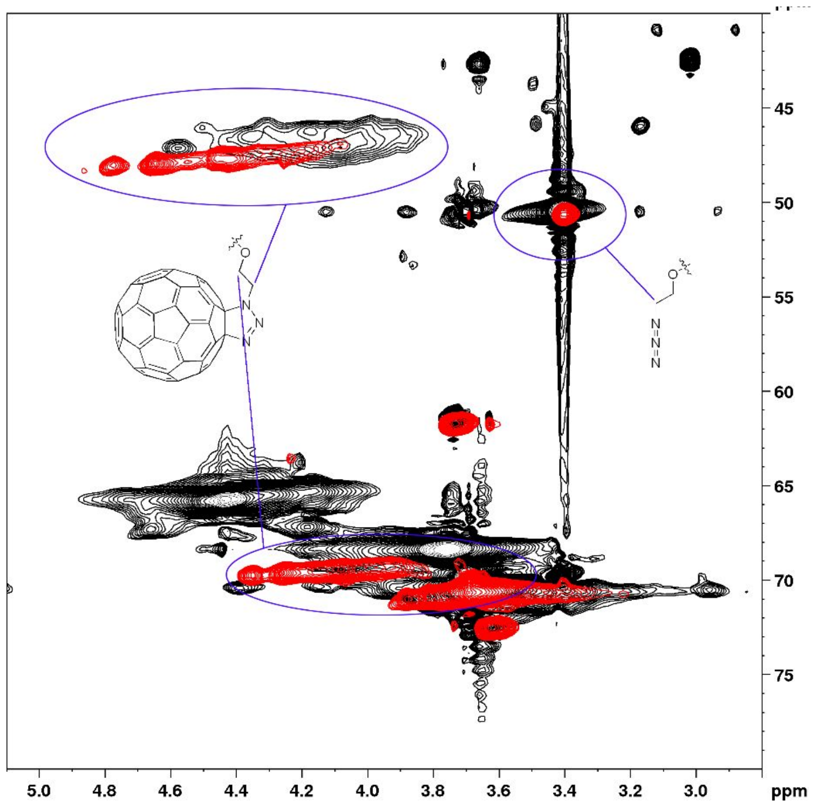

Figure S9. ^{1}H-^{13}C- HSQC spectra of **2** (black) and *ad hoc*-synthesized triazolinofullerene (red).

Ad hoc-synthesized triazolinofullerenes

Buckminster fullerene C_{60} (25 mg, 1 eq.) was dissolved in dry and degassed *o*-dichlorobenzene (5 mL) and 2-(2-(2-(2-azidoethoxy)ethoxy)ethoxy)ethanol (76 mg, 10 eq.) was added at room temperature.[3] The mixture was stirred 30 h at 50 °C under argon, and washed with brine (5 ml × 2). The organic layer was separated, dried over Na_2SO_4, filtered, and evaporated to dryness. The residue was analyzed by HRMS (ESI-TOF): For **5**: HRMS (ESI-TOF) m/z: $[M+Na]^+$ requires 1181.2330, found 1181.1845. For **6**: HRMS (ESI-TOF) m/z: $[M+Na]^+$ requires 1400.35, found 1401.30. For **7,** HRMS (ESI-TOF) m/z: $[M+Na]^+$ requires 1619.47, found 1620.42. This *ad hoc*-prepared mixture of triazolinofullerenes was used as a reference for the NMR measurements (**Figure S7** and **Figure S8**).

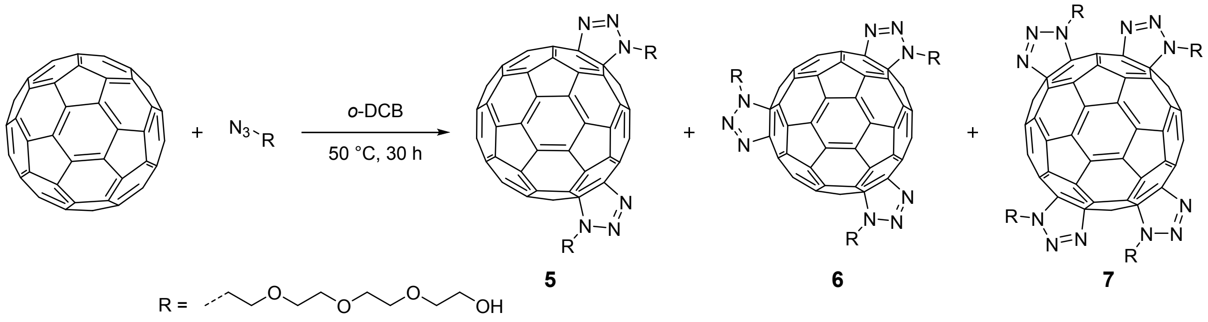

Scheme S1. *Ad hoc*-synthesis of triazolino fullerenes.

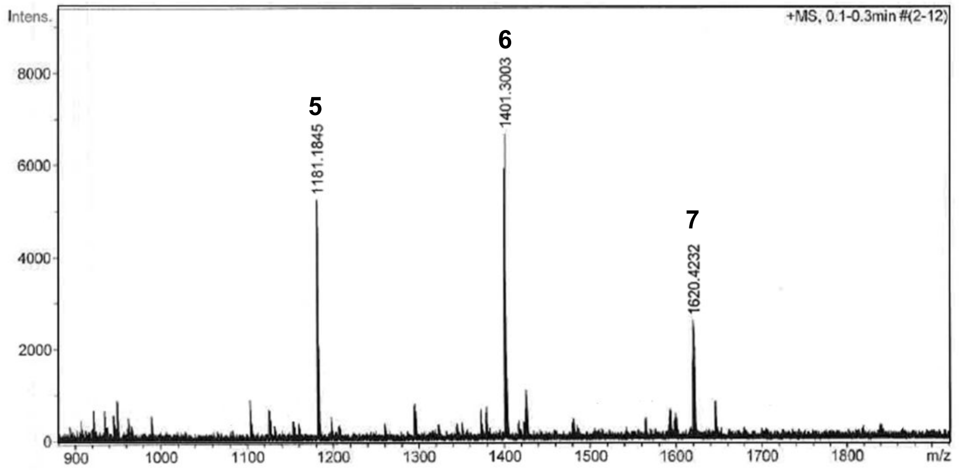

Figure S10. MS-ESI (TOF) analysis of *ad hoc*-synthesized triazolinofullerenes

Synthesis of C_{60}-SNAs S1-S6

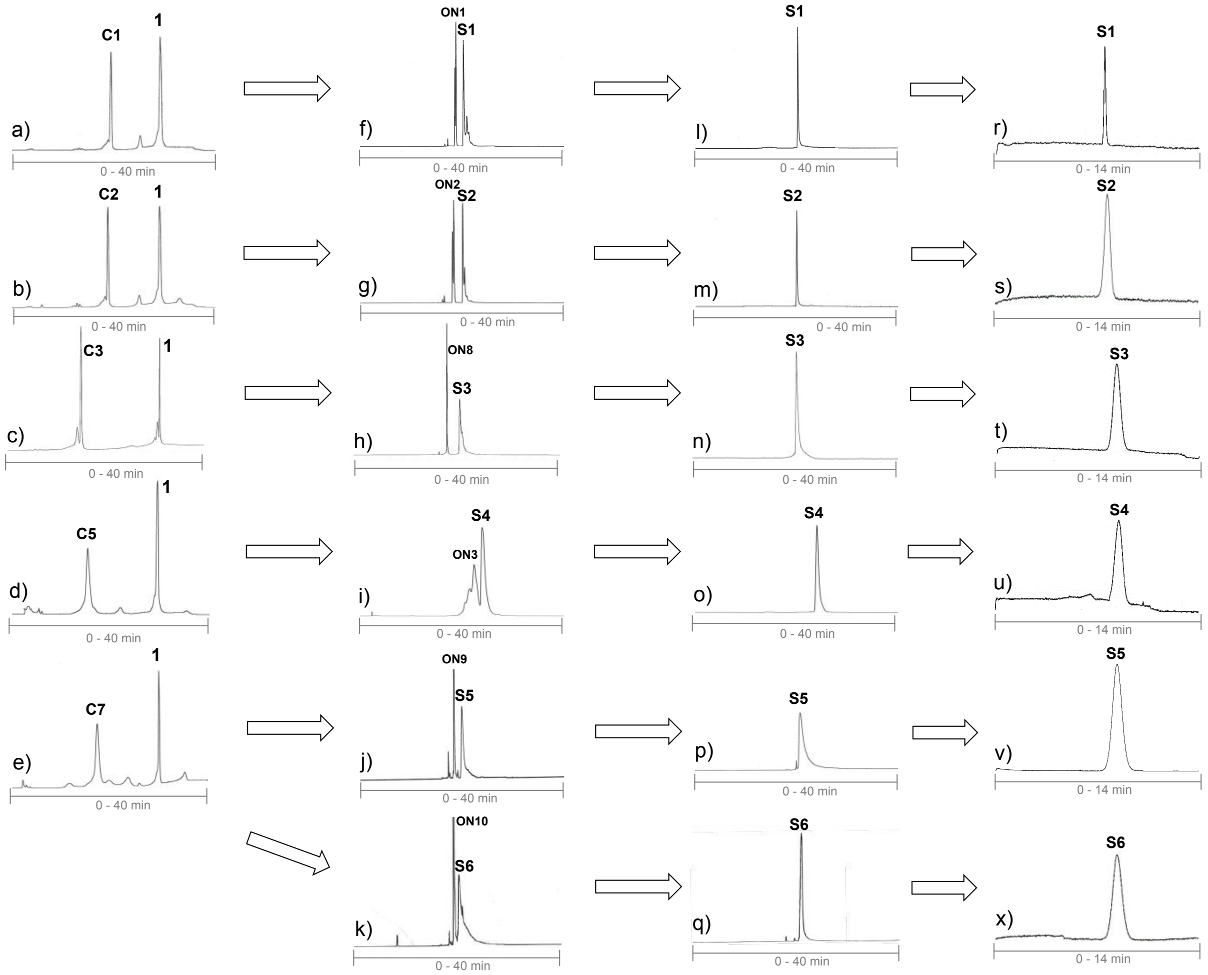

Scheme S2. Purification scheme of the SNAs' assembly. a-e) RP HPLC profiles of crude C_{60}-ON conjugates **C1**-**C7** mixtures), f-k) RP HPLC profiles of crude C_{60}-SNAs **S1**-**S6** mixtures, l-q) RP HPLC profiles of purified C_{60}-SNAs **S1**-**S6**. Capillary electrophoregrams (CE) of purified SNAs **S1**-**S6**. Conditions: a-e) an analytical RP-HPLC column (250 × 4.6 mm, 5 µm), a gradient elution from 40 to 100% MeCN in 50 mmol L^{-1} triethylammonium acetate over 30 minutes and continued then with MeCN, flow rate of 1.0 mL min $^{-1}$, detection at λ = 260 nm, f-q) an analytical RP-HPLC column Phenomenex, Aeris ™ 3.6 µm WIDEPORE XB-C18 200 Å, 150 X 4.6 mm, a linear gradient from 5% to 60% MeCN in 50 mmol L $^{-1}$ triethylammonium acetate over 40 minutes, flow rate of 1.0 mL min $^{-1}$, detection at λ = 260 nm, r-x) capillary zone electrophoresis in a fused silica capillary of 75 µm i.d. and 57 cm effective length, the background electrolyte: 0.3 M citrate buffer, pH 3.1, the voltage of 15 kV and pressure of 0.3 PSI were applied, detection at λ= 260 nm.

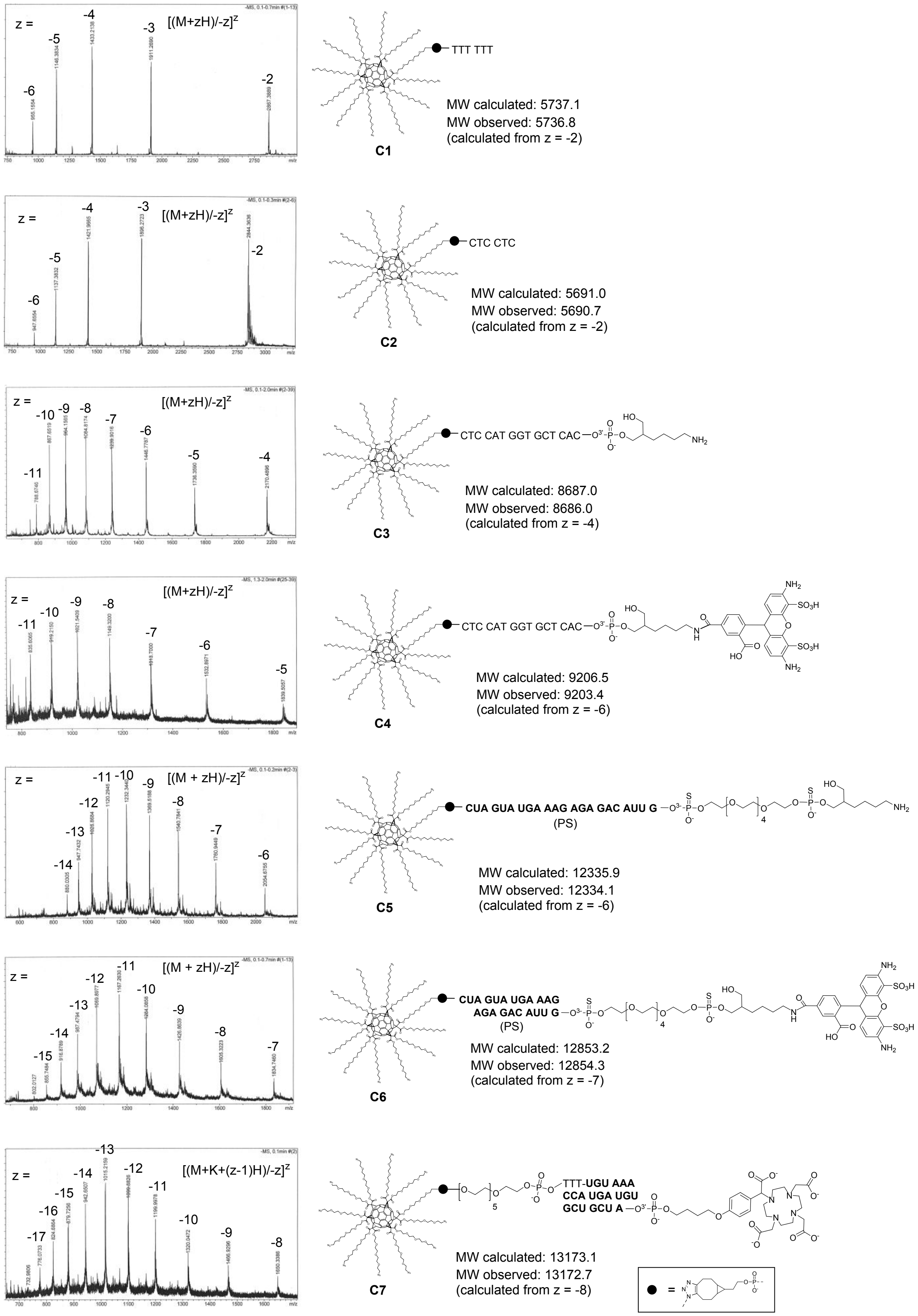

Figure S11. MS(ESI-TOF) data of C_{60}-ON-conjugates (**C1**-**C7**).

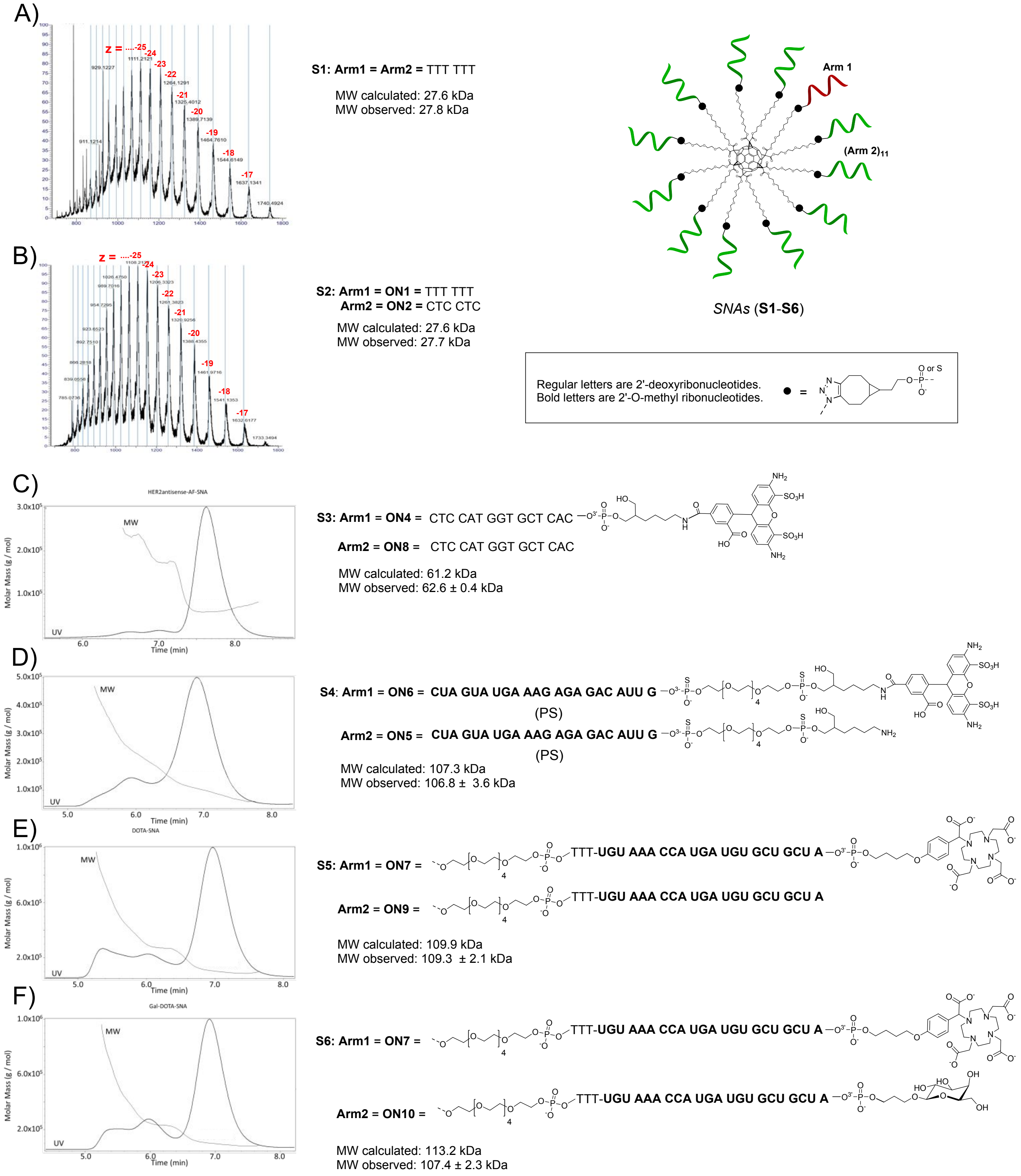

Figure S12. A and B) MS-ESI data of C_{60}-SNAs **S1** and **S2**. C-F) SEC-MALS data of C_{60}-SNAs **S3-S6**.

References

[1] Wrackmeyer, B, *Z. Naturforsch.* **2011,** *66b,* 1079 – 1082.
[2] Banert, K.,Lehmann, J., Quast, H., Meischner, G., Regnat, D., Seiferling, B. *J. Chem. Soc., Perkin Trans. 2,* **2002,** 126 – 134.
[3] Ikuma, N., Mikie, T., Doi, Y., Nakagawa, K., Kokubo, K., Oshima, T., *Org. Lett.* **2012,** *14,* 6040 – 6043.
[4] Sinyashin, O. G., Romanova, I. P., Yusupova, G. G., Nafikova, A. A., Kovalenko, V. I., Azancheev, N. M., Fattakhov, S. G., Reznik, V. S., *Russ. Chem. Bull., Int. Ed.,* **2001,** *50,* 2162 – 2171.
